# Supplementary material for: Signaling by a bacterial phytochrome histidine kinase involves a conformational cascade reorganizing the dimeric photoreceptor
Source: Nat Commun. 2024 Aug 10;15:6853. doi: 10.1038/s41467-024-50412-y (PMC11316798; doi:10.1038/s41467-024-50412-y)
Supplement: Supplementary file 1 — Supplementary Information [file 41467_2024_50412_MOESM1_ESM.pdf]

Supplementary Information

Signaling by a Bacterial Phytochrome Histidine Kinase Involves a Conformational Cascade Reorganizing the Dimeric Photoreceptor

E. Sethe Burgie, Katherine Basore, Michael J. Rau, Brock Summers, Alayna J. Mickles, Vadim Grigura, James A. J. Fitzpatrick, and Richard D. Vierstra

Supplementary Table 1. Cryo-EM Data Collection, Refinement, and Validation Statistics for the *P. syringae* BphP1 Dimer

Photostate	Pr		Pfr			
**Data collection**						
Number of grids used	2		2			
Grid type	Quantifoil 2,2		Quantifoil 2,2			
Microscope/detector	Titan Krios / Falcon 4		Titan Krios / Falcon 4			
Voltage (kV)	300		300			
Magnification	96,000		96,000			
Pixel size (Å)	0.657		0.657			
Total dose ($e^-/Å^2$)	51.78		50.54-51.78			
Frame rate (frames/s)	11.45		11.45			
Dose rate	12.1		11.8-12.1			
Number of frames	49		49			
Total exposure time (s)	4.28		4.28			
Number of micrographs	9,173		12,593			
**Number of particles**						
Picked	3,233,515		4,796,622			
After 2D classification	1,151,696		1,029,141			
**Special identifier**	Pr	Pr	Dimer of Dimers	Dimer of Dimers	Medial	Splayed
**Map boundary[a]**	PSM	extended DHp	FL	PSM	PSM	PSM
**PDB code**	8U4X	8U8Z	8U62	8U63	8U64	8U65
**EMDB code**	41903	42030	41941	41942	41943	41944
**Number of Particles**						
After 3D classification	833,049		402,598	402,598	281,451	232,298
After C2 symmetry expansion	1,666,098		N/A	805,196	562,902	464,596
Final	1,666,098	119,594	402,598	805,196	562,902	464,596
**Map resolution (FSC 0.143)**	2.81	3.45	3.30	3.01	3.13	3.04
**Composition (#)**						
Chains	2	2	2	2	2	2
Non-Hydrogen Atoms	7,873	8,147	7,884	7.758	7,674	7,626
Protein Residues	999	1036	998	984	970	967
Water	0	0	0	0	0	0
Ligands[b]	2	2	2	2	2	2
**Bonds (RMSD)**						
Length (Å)	0.002	0.003	0.003	0.003	0.003	0.002
Angles (°)	0.558	0.654	0.613	0.558	0.589	0.584
**MolProbity score**	1.83		1.58	1.60	1.95	1.93
**Clash score**	9.77	10.48	8.41	8.88	11.26	12.11
**Ramachandran plot (%)**						
Outliers	0.00	0.00	0.00	0.00	0.00	0.00
Allowed	2.83	2.34	2.64	2.67	5.53	4.83
Favored	97.17	97.66	97.36	97.33	94.47	95.17
**Ramachandran Z-score**						
Whole, RMSD	0.85	0.97	0.96	-0.74	-0.22	-0.65
**Peptide plane (#)**						
Cis proline[c]/general	2/0	2/0	2/0	2/0	2/0	2/0
**Mean ADP (B-factors)**						
Protein	63.75	80.90	83.26	68.25	70.71	68.93
Ligand	56.12	52.59	68.42	49.45	47.85	56.67
**Map vs Model**						
CC (mask)	0.79	0.79	0.77	0.79	0.74	0.67
CC (box)	0.78	0.72	0.73	0.77	0.75	0.71
CC (peaks)	0.77	0.67	0.69	0.77	0.73	0.67
CC (volme)	0.79	0.78	0.77	0.80	0.75	0.67
Mean CC for ligands	0.80	0.79	0.76	0.79	0.78	0.64

[a]PSM and FL indicate the portion of the map that was averaged.
[b](LBV) Each protomer included a BV moiety linked by a thioether bond to Cys16
[c]Pro232

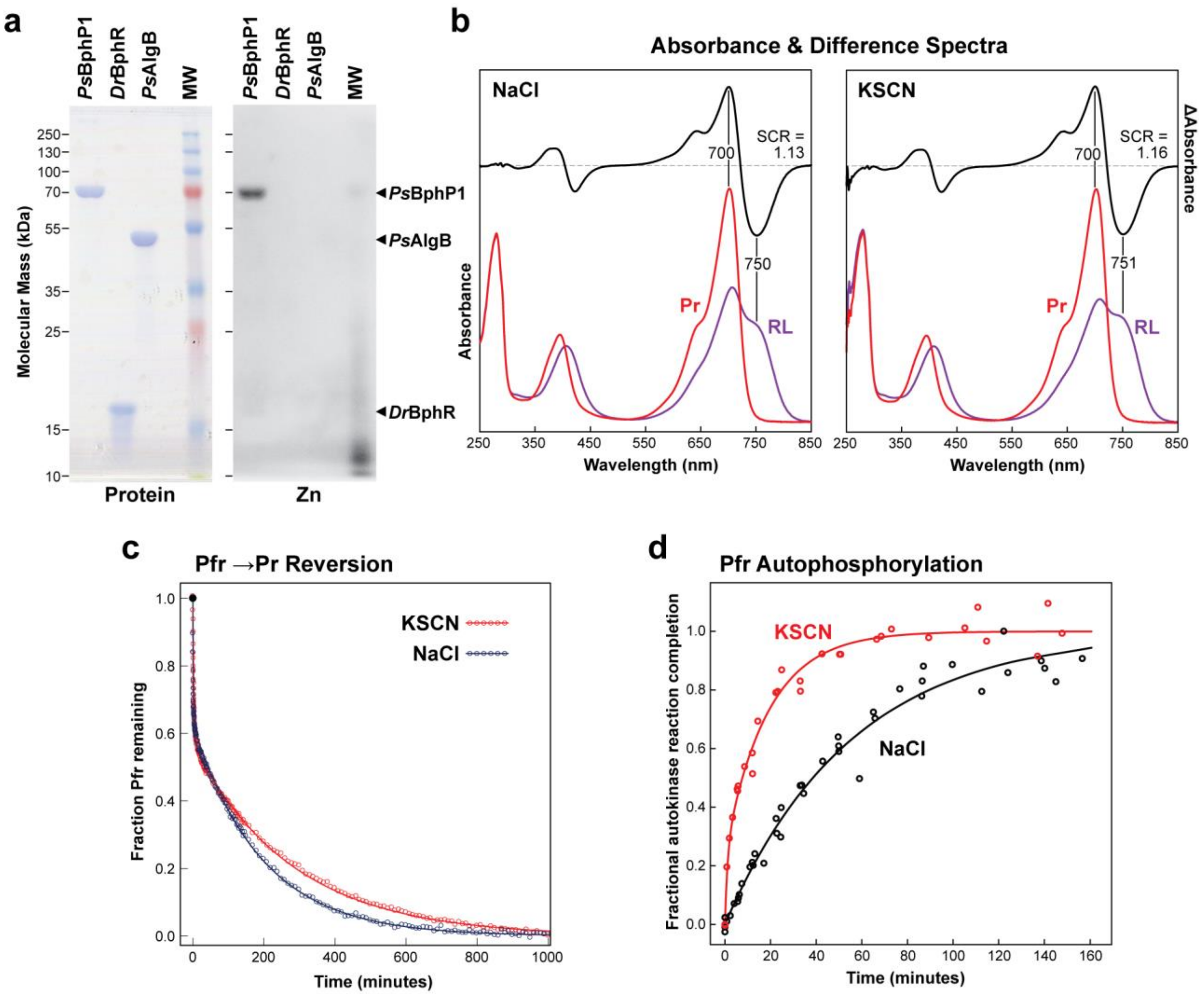

Supplementary Fig. 1. Use of a KSCN-containing buffer did not appreciable alter the spectral and phosphotransferase activities of *Ps*BphP1. **(a)** SDS-PAGE of purified *Ps*BphP1 assembled with BV, along with two response regulators known to work in BphP transmitter kinase cascades - *D. radiodurans* BphR, and *P. syringae* AlgB. The gels were either stained for protein with Coomassie blue (left panel) or imaged for the bound BV by zinc-induced fluorescence under UV light (right panel). **(b)** Comparison of the UV-vis absorbance and difference spectra of *Ps*BphP1 in a buffer containing 175 mM NaCl or 175 mM KSCN. Spectra were collected from dark-adapted samples (Pr) or after saturating irradiation with 630-nm red light (RL, mostly Pfr). Absorbance maxima were determined from the difference spectrum and shown at 70% amplitude. Spectral change ratio (SCR; $-\Delta A_{700nm}/\Delta A_{750nm}$). **(c)** Pfr→Pr thermal reversion of *Ps*BphP1 in buffer containing 175 mM NaCl or 175 mM KSCN. Samples were photoconverted to Pfr with saturating red light and allowed to revert back to Pr in darkness at 22°C. **(d)** Autophosphorylation activity of *Ps*BphP1 is only marginally influenced by a buffer containing 175 mM KSCN versus 175 mM NaCl. *Ps*BphP1 as Pfr was incubated for various times at 22°C with 0.15 mM ATP spiked with [δ-^{32}P]-

ATP, quenched with SDS-PAGE sample buffer, and measured for ^{32}P incorporation by autoradiography of the SDS-PAGE gels. Shown is the combined kinetics from three (KSCN) and four (NaCl) separate experiments. Lines show the best fits to the data.

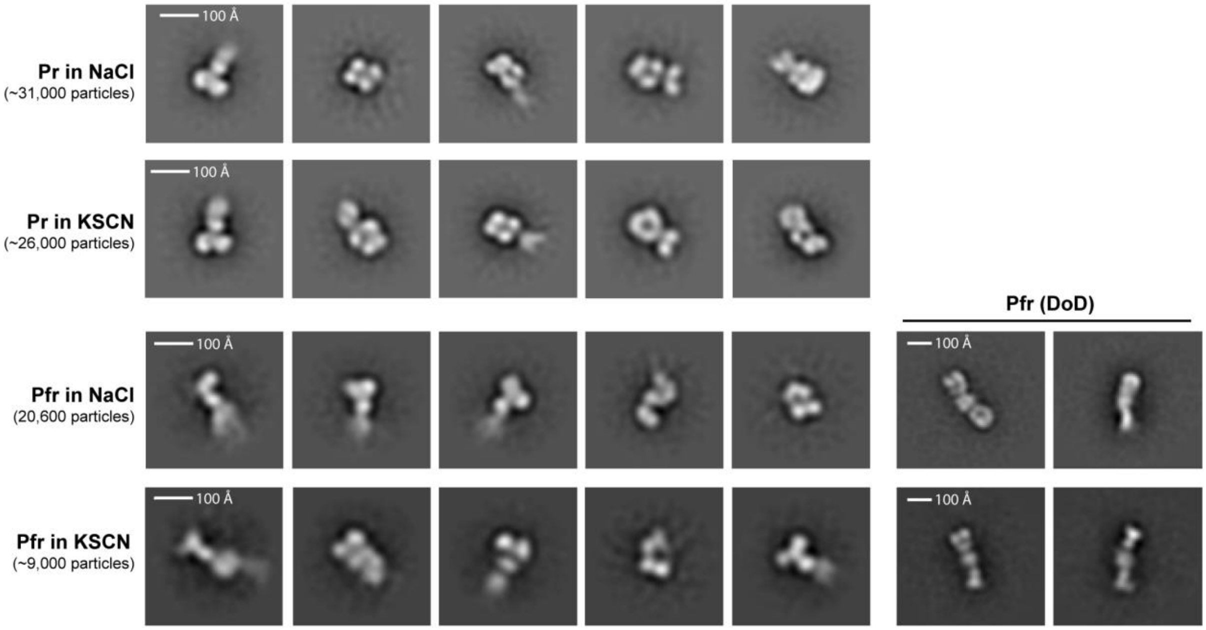

Supplementary Fig. 2. Use of a KSCN- instead of a NaCl-containing buffer did not detectably influence the 3D structure of the *Ps*BphP1 dimer. Full-length *Ps*BphP1 was dissolved in a buffer containing either 175 mM KSCN or 175 mM NaCl, either kept as Pr or photoconverted to Pfr with saturating 630-nm light, and then processed for 2D analysis by negative stain EM. Shown are samplings of 2D class averages for Pr and Pfr. Representative DoD class averages are shown on the right. The number of particles imaged is indicated.

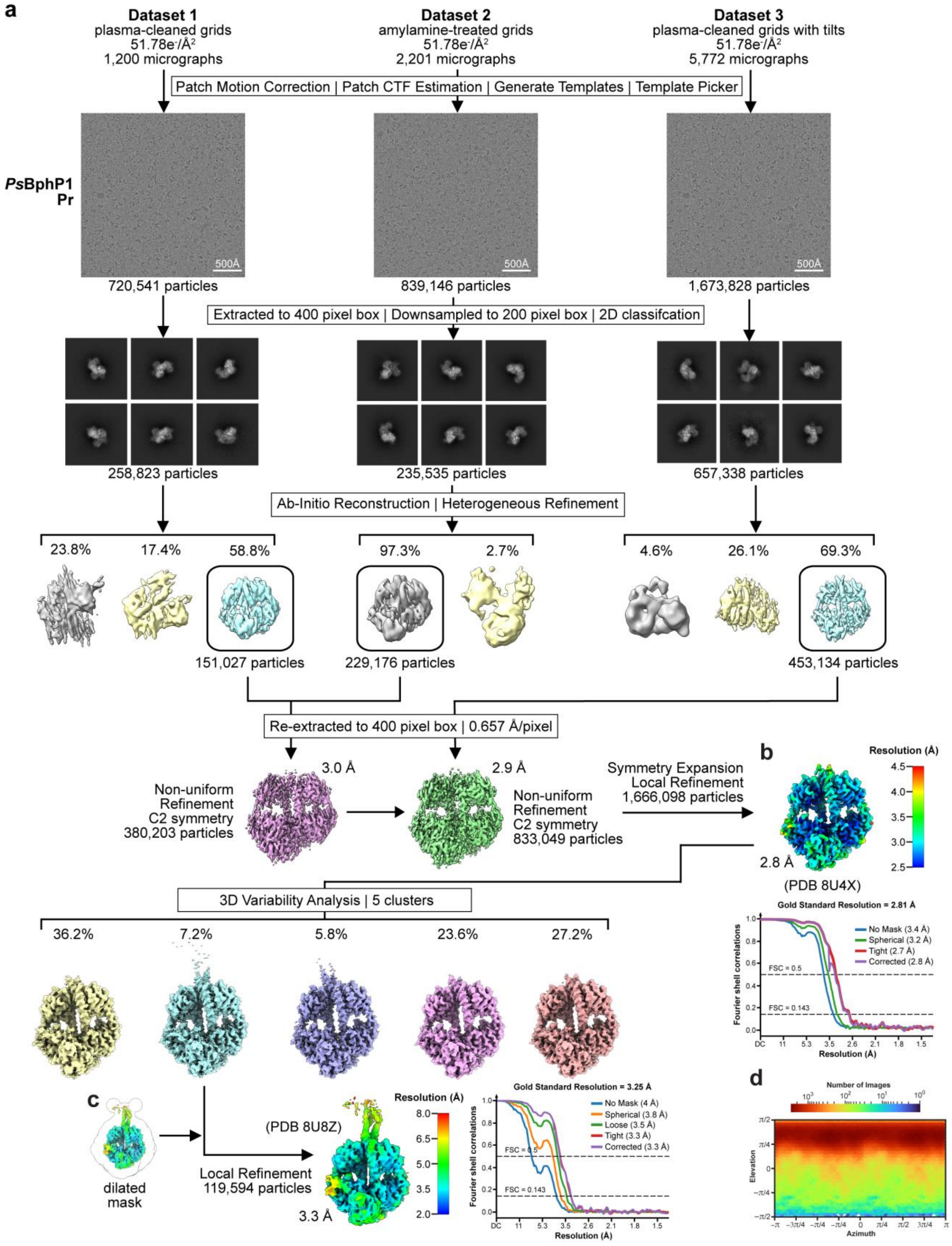

Supplementary Fig. 3. Workflow used for data processing and analysis of the cryo-EM images of the full-length *Ps*BphP1 dimer as Pr. (a) Flow chart used to generate the cryo-EM

model of the PSM starting with 3,233,515 particles. Shown are representative cryo-EM micrographs sampled from plasma-cleaned grids (1,200 micrographs), amylamine-treated grids (2,201 micrographs), and plasma-cleaned grids with tilts (5,772 micrographs). Samplings of 2D class averages, and 3D class averages generated by each set of micrographs are included**.** **(b)** Non-uniform refinement and C2 symmetry analysis generated a 2.9-Å map which was further improved to 2.8-Å resolution by local refinement and C2 symmetry expansion (1,666,098 matching protomers) (PDB 8U4X). (**c**) 3DVA followed by local refinement generated a 3.3-Å map for 119,594 particles that included additional residues within the DHp domains (PDB 8U8Z). Shown is the unsharpened final map with the dilated mask that was used for 3DVA, local refinement, and local resolution estimation. The mask had an additional soft padding width of 20 pixels. The final maps were colored-coded based on the local resolution. Gold-standard Fourier shell correlation (FSC) curves were generated after applying either no mask (blue), a loose spherical mask (green), or a tight mask (red) to both half maps before calculating the FSC. The corrected FSC curves (purple) were calculated using the tight mask with a corrected high-resolution noise substitution. **(d)** Orientation distribution plot of the final local refinement. PDB ID codes for the polished models are indicated.

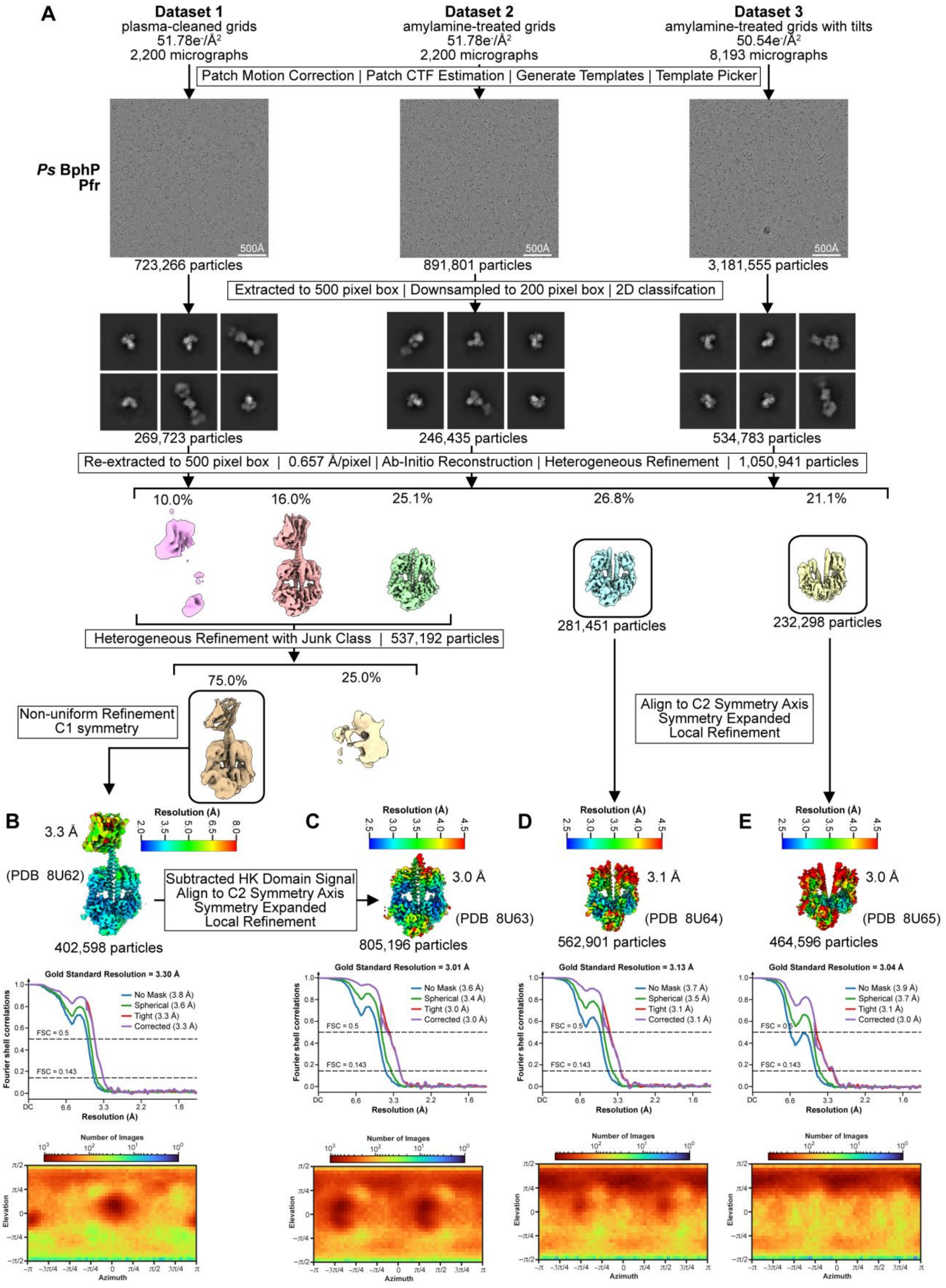

Supplementary Fig. 4. Workflow used for data processing and analysis of the cryo-EM

images of the full-length *Ps*BphP1 dimer as Pfr. (a) Flow chart used to generate the cryo-EM models of the PSM starting with 3,233,515 particles. Shown are representative cryo-EM micrographs sampled from plasma-cleaned grids (2,200 micrographs), amylamine-treated grids (2,200 micrographs), and plasma-cleaned grids with tilts (8,193 micrographs). Samplings of 2D class averages, and 3D class averages generated by each set of micrographs are included. **(b-e)** The resultant maps for the DoDs **(b)**, the PSM derived from DoD **(c)**, and the medial **(d)** and splayed forms **(e)** (PDB ID codes 8U62, 8U63, 8U64, and 8U65, respectively). Included are the maps colored by local resolution (*top*), and the corresponding Gold-standard Fourier shell correlation (FSC) curves (*middle*) and orientation distribution plots (*bottom*). FSC curves were generated after applying either no mask (blue), a loose spherical mask (green), or a tight mask (red) to both half maps before calculating the FSC. The corrected FSC curves (purple) were calculated using the tight mask with a corrected high-resolution noise substitution.

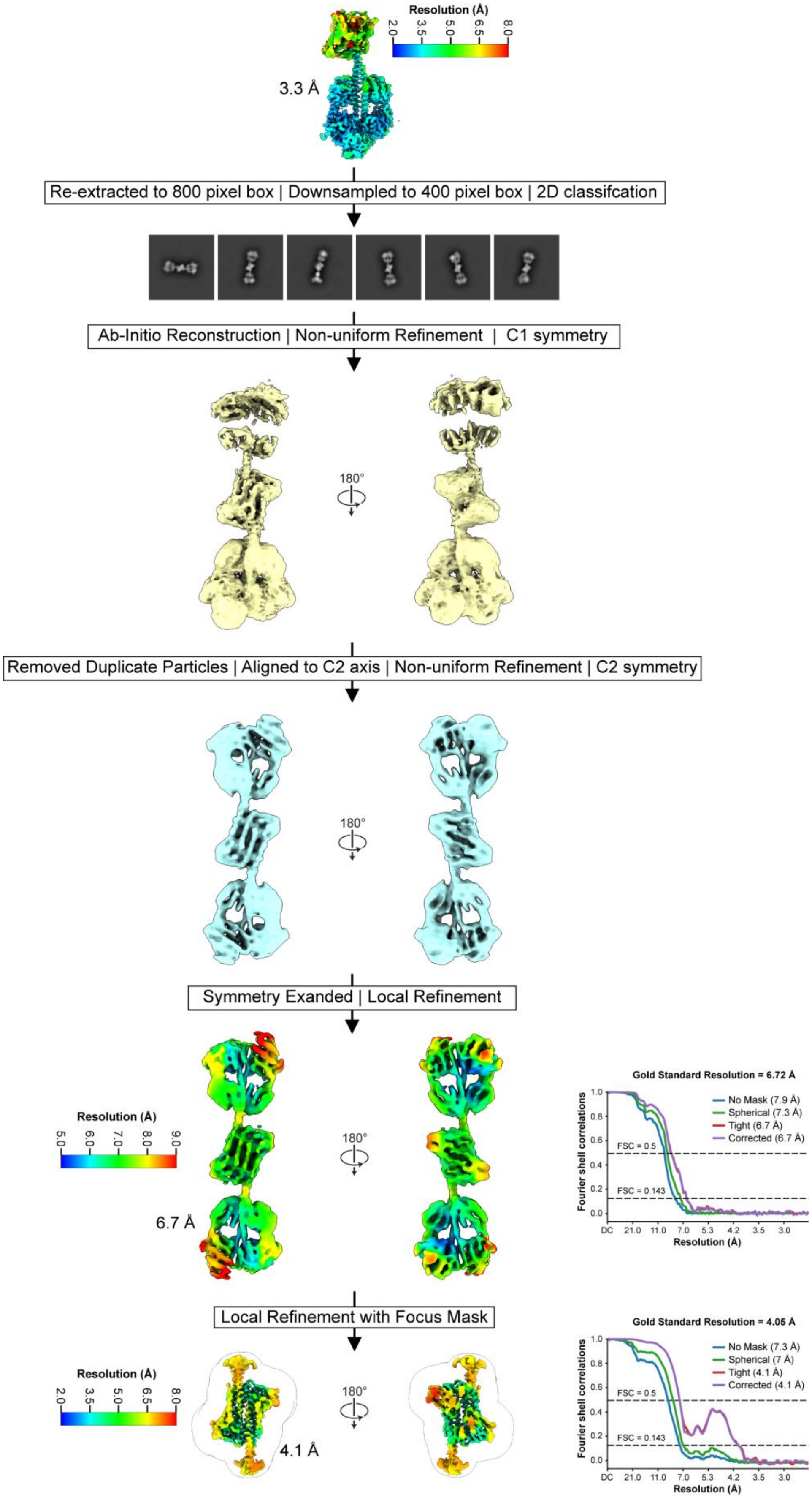

Resolution (Å)
2.0 3.5 5.0 6.5 8.0
3.3 Å
Re-extracted to 800 pixel box | Downsampled to 400 pixel box | 2D classifcation
Ab-Initio Reconstruction | Non-uniform Refinement | C1 symmetry
180°
Removed Duplicate Particles | Aligned to C2 axis | Non-uniform Refinement | C2 symmetry
180°
Symmetry Exanded | Local Refinement
Resolution (Å)
5.0 6.0 7.0 8.0 9.0
180°
6.7 Å
Gold Standard Resolution = 6.72 Å
No Mask (7.9 Å)
Spherical (7.3 Å)
Tight (6.7 Å)
Corrected (6.7 Å)
FSC = 0.5
FSC = 0.143
Fourier shell correlations
Resolution (Å)
DC 21.0 11.0 7.0 5.3 4.2 3.5 3.0
Local Refinement with Focus Mask
Resolution (Å)
2.0 3.5 5.0 6.5 8.0
180°
4.1 Å
Gold Standard Resolution = 4.05 Å
No Mask (7.3 Å)
Spherical (7 Å)
Tight (4.1 Å)
Corrected (4.1 Å)
FSC = 0.5
FSC = 0.143
Fourier shell correlations
Resolution (Å)
DC 21.0 11.0 7.0 5.3 4.2 3.5 3.0

Supplementary Fig. 5. Workflow used for data processing and analysis of the cryo-EM images for the DoD subpopulation of *Ps*BphP1 generated as Pfr. Starting with the particle stack corresponding to the 3.3-Å map for the DoD (colored by local resolution), particles were re-extracted to a 800-pixel box, downsampled to 400 pixels (for a pixel size of 1.314 Å) and underwent a round of 2D classification to visualize the tetrameric arrangement. A new model was generated *ab-initio* and refined with no applied symmetry. Duplicate particles were removed with a minimum separation distance of 250 Å. Then both the particles and map were recentered in the box and aligned so that the C2 symmetry axis was perpendicular to the helices of the DHp domains. After refinement with C2 symmetry applied, the particles were symmetry expanded and underwent local refinement, resulting in a 6.7-Å map. To further refine the DHp domains, a focused mask was applied during a subsequent round of local refinement. The dilated mask shown had an additional soft padding width of 10 pixels. Resolution anisotropy was noticed, as evidenced by the increased correlation bump at higher resolution, and was likely generated by the preferred orientations of the particles. The final map at 4.1-Å resolution was colored-coded based on the local resolution. FSC curves were generated after applying either no mask (blue), a loose spherical mask (green), or a tight mask (red) to both half maps before calculating the FSC. The corrected FSC curves (purple) were calculated using the tight mask with a corrected high-resolution noise substitution.

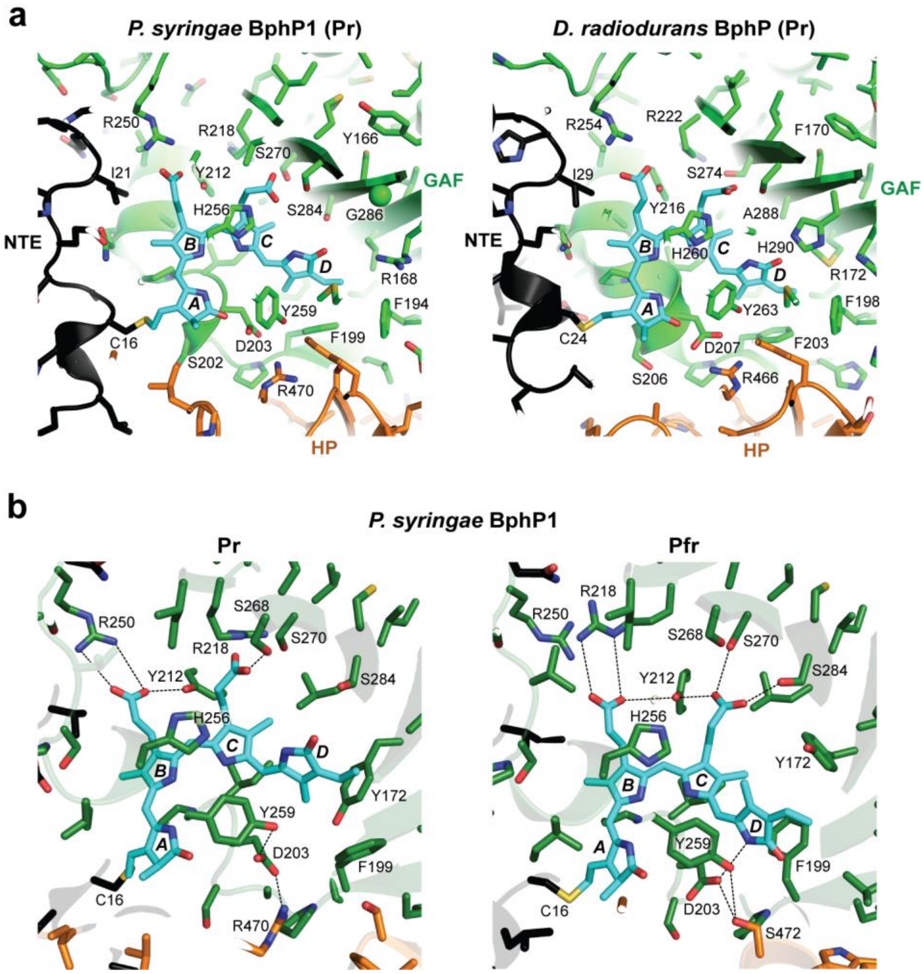

Supplementary Fig. 6. 3D structure of the BV-binding pocket of the full-length *Ps*BphP1 dimer as Pr and Pfr. (a) Comparison of the pocket as Pr from *Ps*BphP1 determined here by cryo-EM (PDB 8U4X) with that determined by X-ray crystallography of the PSM from *D. radiodurans* (*Dr*) BphP (PDB 4Q0J[14]). **(b)** Comparison of the pocket from *Ps*BphP1 as Pr (PDB 8U4X) and Pfr (PDB 8U63). The A-D pyrrole rings (cyan) of BV are labelled. Possible hydrogen bonds are shown by the dashed lines. The NTE, GAF, and PHY hairpin features are colored in black, green, and orange, respectively. Yellow highlights the sulfur atom in the Cys16 used to form the thioether linkage with BV.

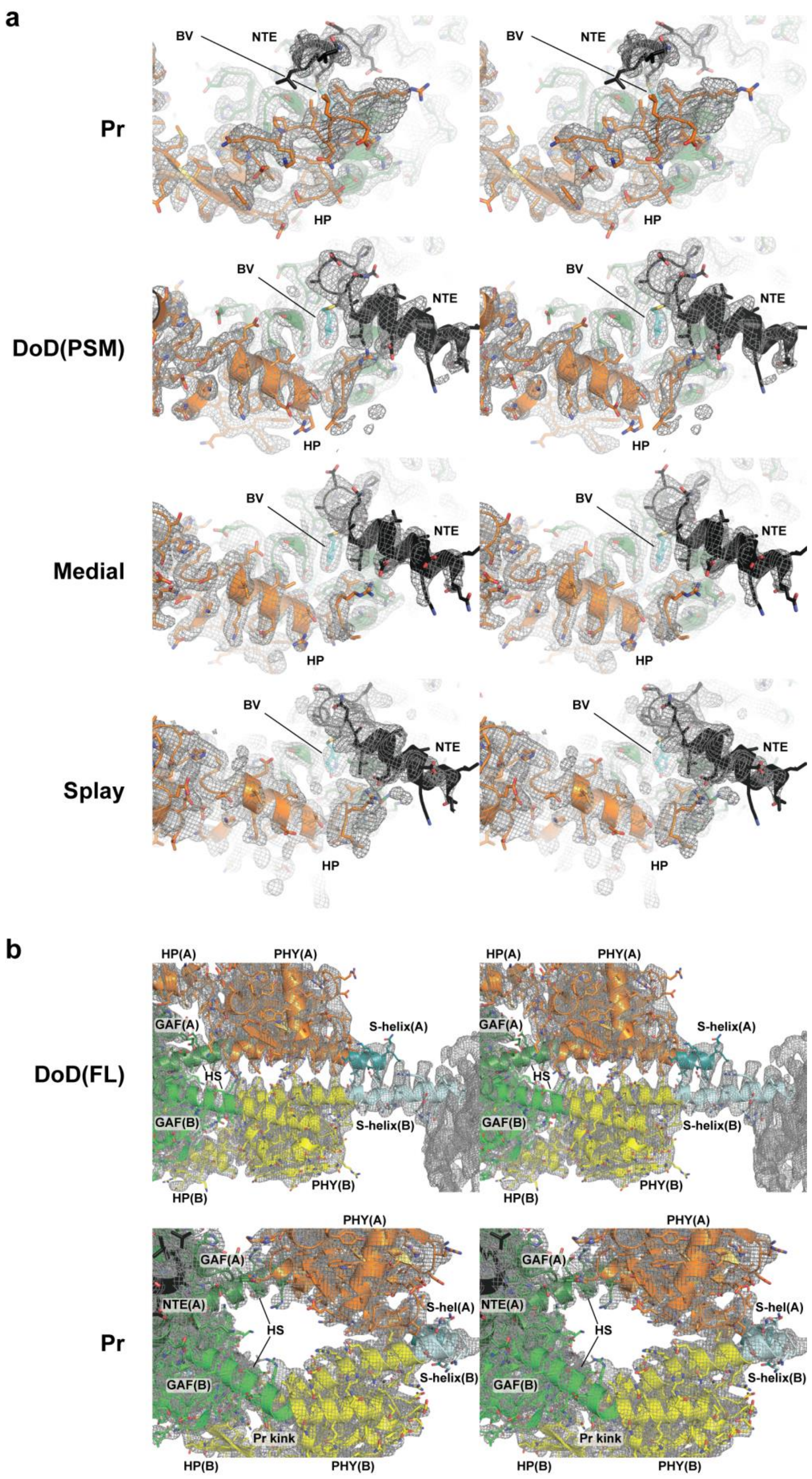

Supplementary Fig. 7. Overlay of the EM density map to the derived model for the *Ps*BphP1 dimer showing the map and model quality. Cartoon views of the GAF and PHY/hairpin domains and the hairpin (HP), helical spine (HS), NTE, S-helix/DHp regions of the two protomers are colored green, orange/yellow, black, and cyan/light blue, respectively. The maps are shown in grey mesh and side chains are shown as sticks. **(a)** Stereoviews of the region encompassing the HP and NTE in the high resolution map of Pr (PDB 8U4X) and the DoD (PDB 8U63), medial (PDB 8U65), and splayed maps (PDB 8U66) of Pfr. Location of BV (cyan) is indicated. **(b)** Stereoviews of the HP, HS, and S-helix

regions in the high resolution map for Pr (PDB 8U4X) and the DoD map for Pfr (PDB 8U62). Cartoon views of the HP and NTE are colored orange and black, respectively.

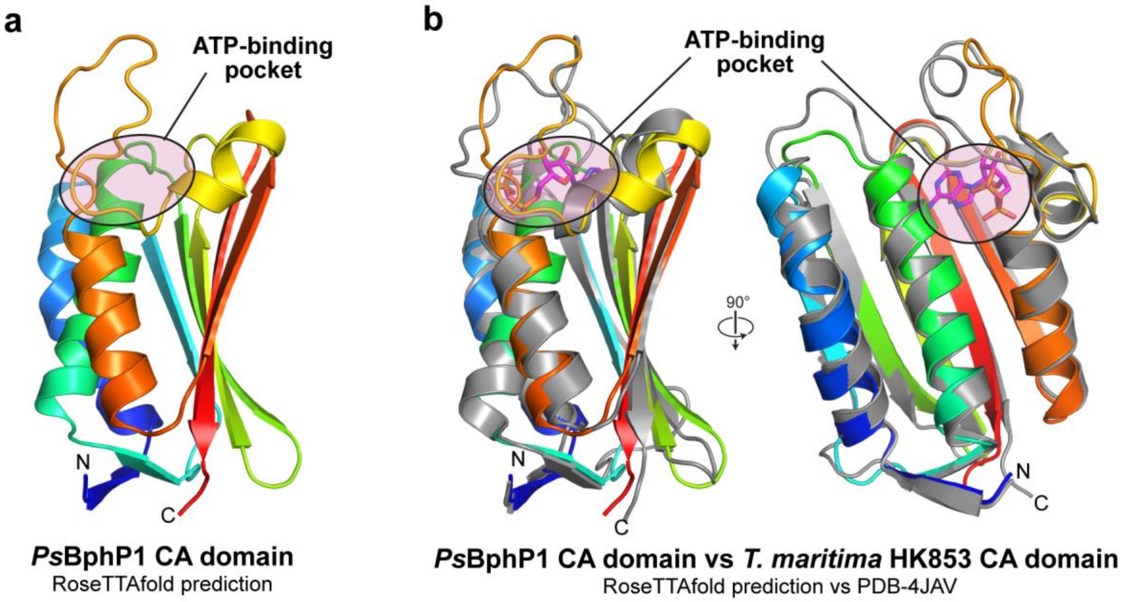

Supplementary Fig. 8. Predicted 3D structure of the CA domain in *Ps*BphP1 determined by RoseTTAFold. (a) Predicted cartoon model of the CA domain oriented in the same position as the rightward CA domain found in Fig. 2c. **(b)** Superposition of the predicted cartoon model of the CA domain (shown in color) with orthogonal views determined by X-ray crystallography for the CA domain from the *Thermotoga maritima* HK853 transmitter kinase bound with ADP (PDB 4JAV[36]) shown in grey. Position of the ADP-binding pocket is highlighted by the pink ovals. N, amino terminus. C, carboxy terminus.

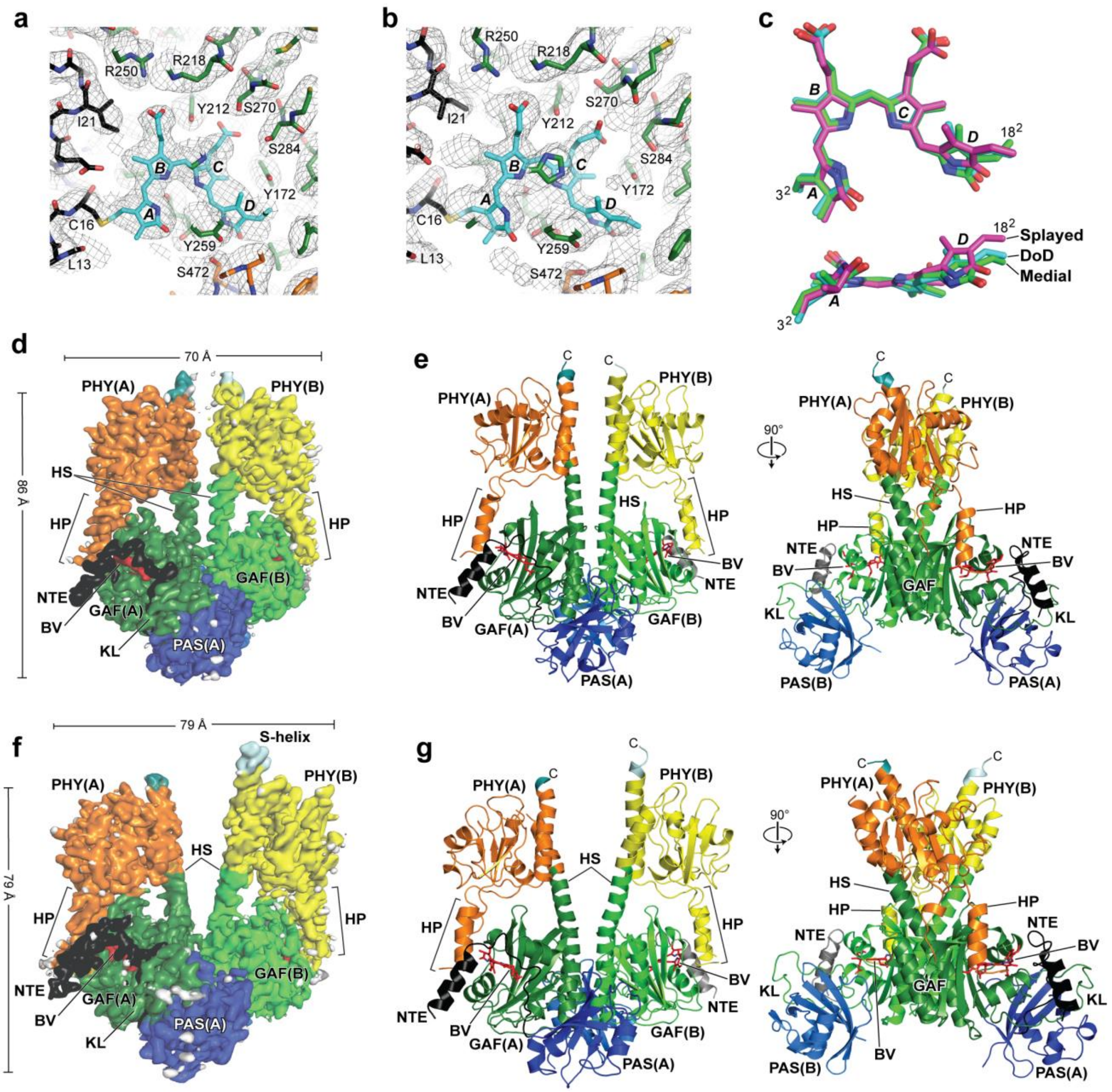

Supplementary Fig. 9. 3D structure of the PSM from the *Ps*BphP1 dimer as Pfr in both medial (PDB 8U64) and splayed (PDB 8U65) configurations. The two maps emerged from C2 symmetry alignment and expansion followed by local refinement of the Pfr particles described in Supplementary Fig. 4. **(a** and **b)** Cryo-EM models of the BV-binding pocket as Pfr from the medial **(a)** and splayed PSM maps **(b)**. In each image, the A protomer (sticks) was superposed with the EM map (grey mesh). The nitrogen, oxygen, and sulfur atoms are in blue, red, and yellow, respectively. The A-D pyrrole rings are labelled. BV and the NTE, GAF domain, and PHY hairpin carbon atoms are in cyan, black, green, and orange, respectively. Cys16 that forms the thioether linkage with BV is labelled. **(c)** Superposed stick models of BV in the three cryo-EM models of Pfr (DoD, cyan; medial, green; and splayed, magenta). The models were aligned based on the

structures of the respective GAF domains. **(d** and **f)** Surface-rendered views of medial **(d)** and splayed 3D EM maps **(f)** of the PSM as Pfr. EM density was rendered at 5 σ. EM density within 3 Å of a modeled atom was color-coded by domain with NTE, PAS, GAF, PHY, S-helix and BV colored in black, blue, green, yellow/orange, teal/light cyan, and red, respectively. EM densities found at distances greater than 3 Å from the nearest atom were rendered white. Positions of the helical spines (HS), HP, NTE, and KL features are highlighted. Dimensions of the dimeric PSM are indicated. **(e** and **g)** Orthogonal cartoon views of the atomic model of the dimeric PSM for the medial **(e)** and splayed maps **(g)**. The various features are colored as in panel (d). BV is shown in red sticks. C, C-terminus.

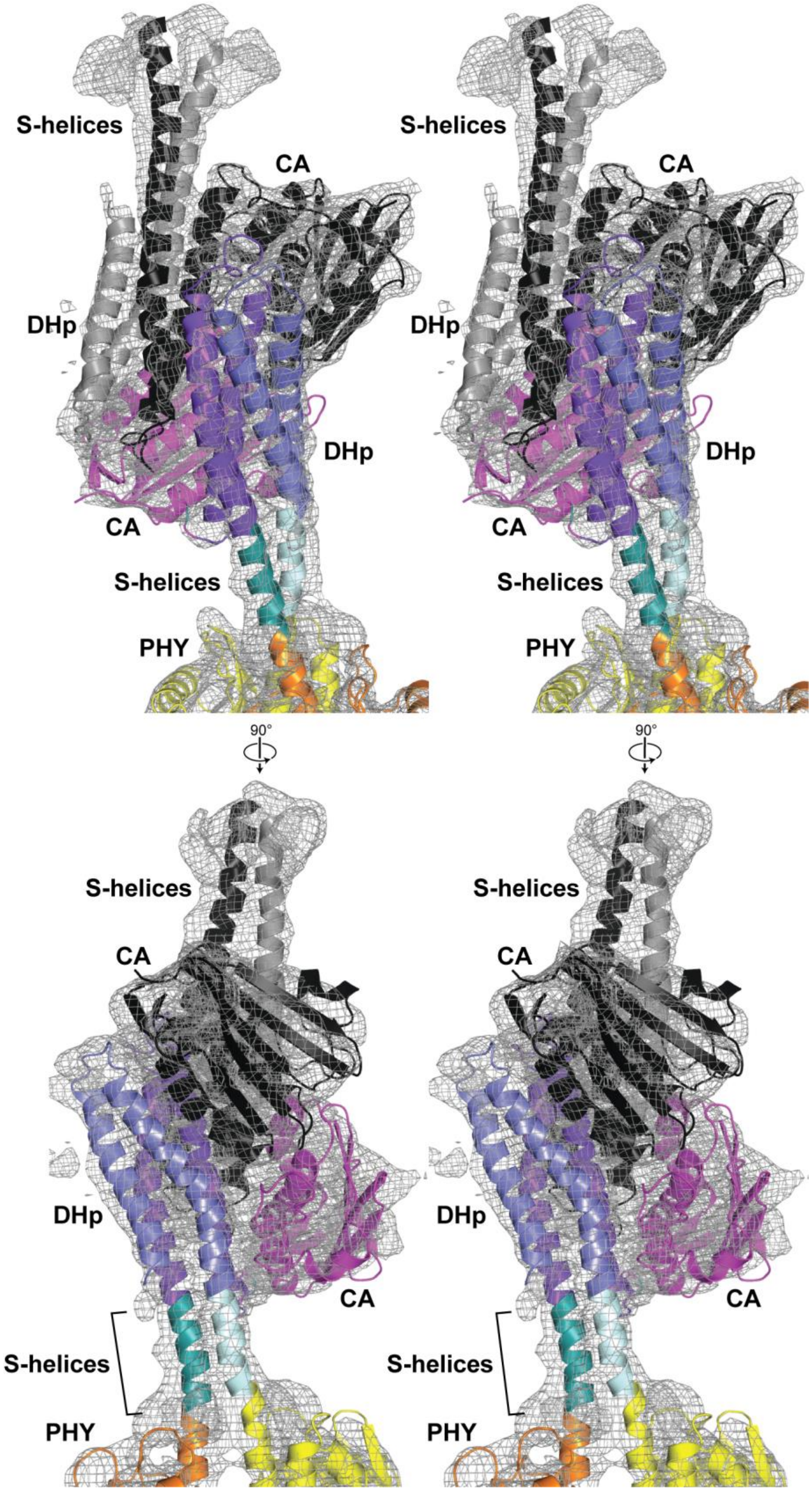

Supplementary Figure 10. Orthogonal stereoviews of the interface between *Ps*BphP1 dimers in the DoD tetramer configuration as Pfr. The model is shown in cartoon and superposed with a composite EM map contoured at 20 σ (grey mesh). PHY, DHp and CA domains, and *S*-helices in subunit A are shown yellow/orange, blue/purple, magenta, and cyan/light blue, respectively. The cartoon features in subunit B (truncated to the end of the *S*-helices) are shown in grey/black.

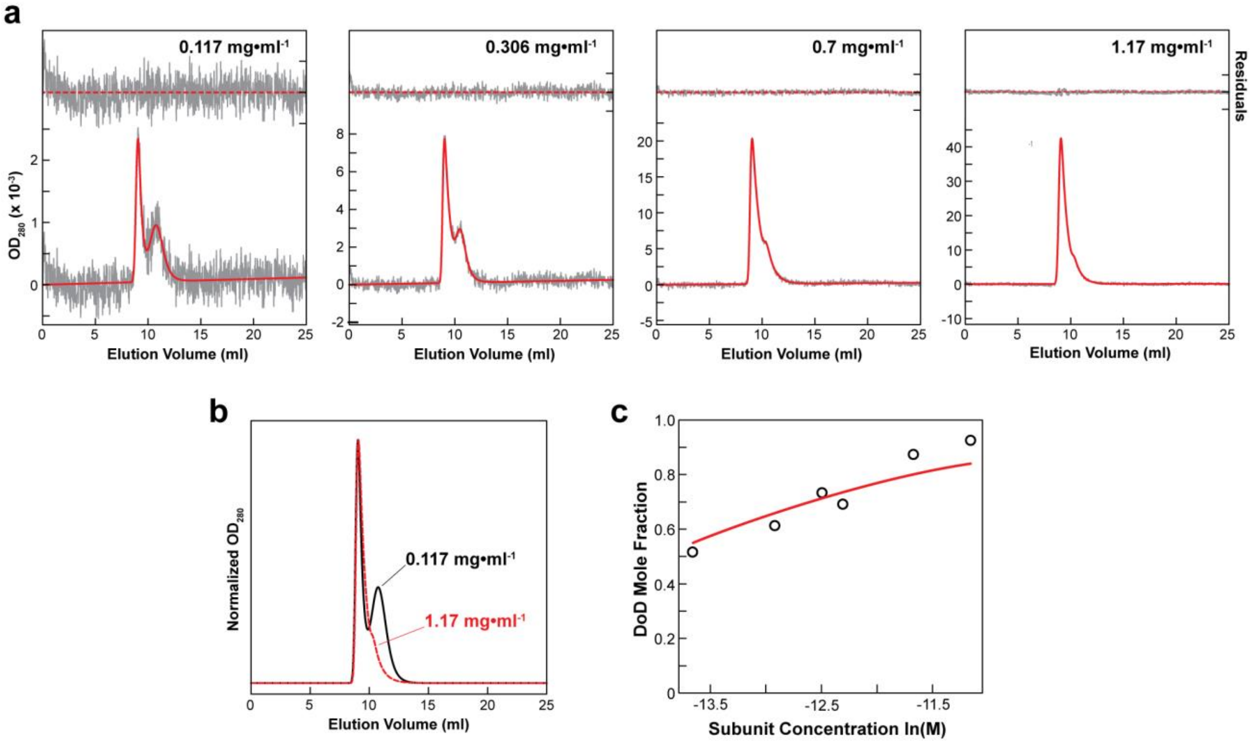

Supplementary Fig. 11. Concentration-dependent assembly of full-length *Ps*BphP1 dimers as Pfr into dimer-of-dimers (DoDs).

Increasing concentrations of *Ps*BphP1 as Pfr were subjected to SEC with the elution monitored at OD_{280}. **(a)** Elution profiles of *Ps*BphP1 at the indicated concentrations. Red lines show the fit of the absorption data to a modified Gaussian curve for two populations plus a regression curve to adjust for baseline drift. Residuals are the difference between the absorption data points and the fit. **(b)** Comparison of the elution profile fits corrected for baseline drift at 0.117 mg mL^{-1} and 1.17 mg mL^{-1} to highlight the elution volume differences between the dimer and DoD particles. Curves were normalized by setting the early (tetramer) elution peak maxima to one. **(c)** Mole fraction of *Ps*BphP1 assembled into DoDs as a function of subunit concentration. The red line is the best fit of the data to a dimer/DoD equilibrium.

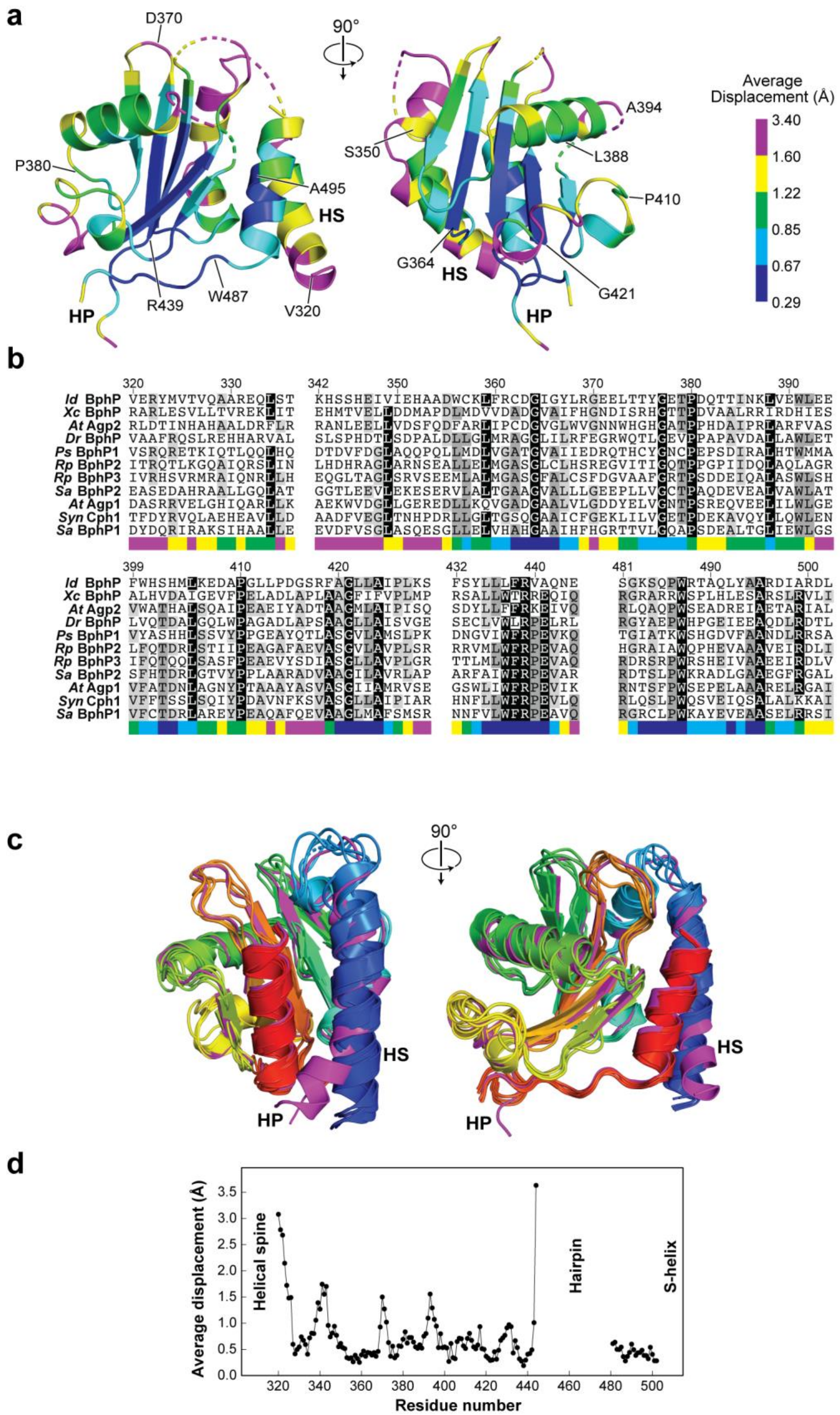

Supplementary Fig. 12. The central core of the PHY domain from BphPs folds into a highly stable structure.

(a) Structural superposition of 51 PHY domains from Phys with available 3D models. The average absolute value displacements from the mean α-carbon position (Å) of the models at a given residue are indicated by the color bar. **(b)** Sequence alignment of PHY domains from representative Phys shown in panel (a). Top, alignment showing the levels of identity and similarity as indicated by the black and grey boxes, respectively. Bottom, average displacement of the residue from the mean. Abbreviations: *Idiomarina A28L*, *Xc*, *Xanthomonas campestris*; *At*, *Agrobacterium tumefaciens*; *Dr*, *Deinococcus radiodurans*; *Rp*, *Rhodopseudomonous palustris*; *Sa*, *Stigmatella aurantiaca*; and *Syn*, *Synechococcus* PCC6803. **(c)** Superposition of the 3D structures of the PHY domain from *P. syringae* BphP determined here for Pr (magenta), and the DoD, medial, and splayed models for Pfr (rainbow heat map). **(d)** Average absolute value displacement from the mean for each amino acid of the four models of the PHY domain from *Ps*BphP1 displayed in panel (c). Positions of adjacent helical spine, hairpin and S-helices are indicated.

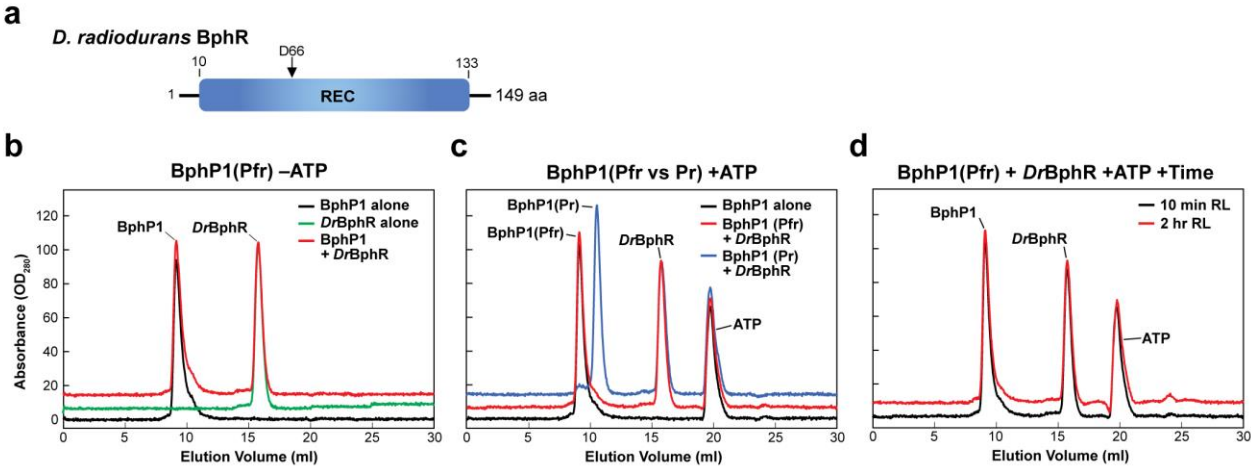

Supplementary Fig. 13. The *D. radiodurans* (*Dr*) response regulator BphR neither interacts with *Ps*BphP1 nor stimulates ATP hydrolysis. **(a)** Domain architecture of *Dr*BphR showing the response receiver (REC) domain. Asp66 is the predicted phosphoacceptor site for transmitter HKs[28]. REC, phosphoacceptor receiver domain. **(b-d)** SEC chromatograms of *Ps*BphP1 as Pr or Pfr with or without *Dr*BphR and/or ATP. Recombinant *Ps*BphP1 (as Pr of Pfr) and/or *Dr*BphR were subjected to SEC with or without 1.5 mM ATP added to the reaction mixture. Elution profile was monitored at OD_{280}. Note that *Ps*BphP1 at the concentration used (3 mg mL^{-1}) assembles as a dimer as Pr but mostly as a DoD as Pfr (see Supplementary Fig. 11). *Dr*BphR was added at a concentration of 3 mg mL^{-1}. **(b)** Profiles of *Ps*BphP1 and *Dr*BphR as Pfr either alone or mixed in the absence of ATP. **(c)** Profiles of *Ps*BphP1 as Pr or Pfr either alone or mixed with *Dr*BphR in the presence of ATP. **(d)** Profiles of *Ps*BphP1 as Pfr mixed with *Dr*BphR in the presence of ATP and irradiated with red light (RL) for 10 min or 2 hr before SEC. See Fig. 6 for similar binding studies of *P. syringae* AlgB mixed with *Ps*BphP1.
