# Supplementary material for: Signaling by a bacterial phytochrome histidine kinase involves a conformational cascade reorganizing the dimeric photoreceptor
Source: Nat Commun. 2024 Aug 10;15:6853. doi: 10.1038/s41467-024-50412-y (PMC11316798; doi:10.1038/s41467-024-50412-y)
Supplement: Supplementary file 3 — Reporting Summary [file 41467_2024_50412_MOESM3_ESM.pdf]

nature portfolio

Corresponding author(s): Dr. Richard D. Vierstra

Last updated by author(s): 6-13-2024

Reporting Summary

Nature Portfolio wishes to improve the reproducibility of the work that we publish. This form provides structure for consistency and transparency in reporting. For further information on Nature Portfolio policies, see our Editorial Policies and the Editorial Policy Checklist.

Statistics

For all statistical analyses, confirm that the following items are present in the figure legend, table legend, main text, or Methods section.

n/a	Confirmed	
☐	☒	The exact sample size (*n*) for each experimental group/condition, given as a discrete number and unit of measurement
☐	☒	A statement on whether measurements were taken from distinct samples or whether the same sample was measured repeatedly
☒	☐	The statistical test(s) used AND whether they are one- or two-sided<br>*Only common tests should be described solely by name; describe more complex techniques in the Methods section.*
☒	☐	A description of all covariates tested
☒	☐	A description of any assumptions or corrections, such as tests of normality and adjustment for multiple comparisons
☒	☐	A full description of the statistical parameters including central tendency (e.g. means) or other basic estimates (e.g. regression coefficient) AND variation (e.g. standard deviation) or associated estimates of uncertainty (e.g. confidence intervals)
☒	☐	For null hypothesis testing, the test statistic (e.g. *F*, *t*, *r*) with confidence intervals, effect sizes, degrees of freedom and *P* value noted<br>*Give P values as exact values whenever suitable.*
☒	☐	For Bayesian analysis, information on the choice of priors and Markov chain Monte Carlo settings
☒	☐	For hierarchical and complex designs, identification of the appropriate level for tests and full reporting of outcomes
☒	☐	Estimates of effect sizes (e.g. Cohen's *d*, Pearson's *r*), indicating how they were calculated

Our web collection on statistics for biologists contains articles on many of the points above.

Software and code

Policy information about availability of computer code

Data collection	Cryo-EM maps were generated with a Titan Krios electron microscope using a Falcon 4 direct electron detector (ThermoFischer Scientific)
Data analysis	EM map densities were acquired from movies motion-corrected with CryoSPARC v4. 2.1 with subsequent CTF estimation of the micrographs. Cryo-EM models were built with Phenix followed by manual model building in COOT. Structural Figures were prepared with UCSF Chimera. UCSF ChimeraX and Pymol.

For manuscripts utilizing custom algorithms or software that are central to the research but not yet described in published literature, software must be made available to editors and reviewers. We strongly encourage code deposition in a community repository (e.g. GitHub). See the Nature Portfolio guidelines for submitting code & software for further information.

Data

Policy information about availability of data

All manuscripts must include a data availability statement. This statement should provide the following information, where applicable:

- Accession codes, unique identifiers, or web links for publicly available datasets
- A description of any restrictions on data availability
- For clinical datasets or third party data, please ensure that the statement adheres to our policy

The 3D cryo-EM map of full-length PsBphP1 as Pr has been deposited in the Electron Microscopy Data Bank database under accession codes EMD-41903. The corresponding atomic model has been deposited in the RCSB Protein Data Bank (http://www.rcsb.org) under accession code PDB-8U4X. The lower resolution map

and model of Pr with an extended view of the DHp domain are available in the Electron Microscopy and RCSB Protein Data Banks under accession codes EMD-42030 and PDB-8U8Z, respectively. The 3D cryo-EM maps of PsBphP1 as Pfr used to generate the full-length DoD, the DoD PSM, the medial PSM, and the splayed PSM models are available in the Electron Microscopy Data Bank database under accession codes EMD-41941, EMD-41942, EMD-4194, and EMD41944, respectively. The corresponding atomic models for the four Pfr maps have been deposited in the RCSB Protein Data Bank under accession codes PDB-8U62, PDB-8U63, PDB-8U64, and PDB-8U65, respectively. The 3D structure of the PSM from DrBphP29 was obtained from the RCSB Protein Data Bank under the accession code PDB 4Q0J. Source data are provided with this paper (Supplementary Dataset-1).

Research involving human participants, their data, or biological material

Policy information about studies with human participants or human data. See also policy information about sex, gender (identity/presentation), and sexual orientation and race, ethnicity and racism.

Reporting on sex and gender	NA
Reporting on race, ethnicity, or other socially relevant groupings	NA
Population characteristics	NA
Recruitment	NA
Ethics oversight	NA

Note that full information on the approval of the study protocol must also be provided in the manuscript.

Field-specific reporting

Please select the one below that is the best fit for your research. If you are not sure, read the appropriate sections before making your selection.

☒ Life sciences ☐ Behavioural & social sciences ☐ Ecological, evolutionary & environmental sciences

For a reference copy of the document with all sections, see nature.com/documents/nr-reporting-summary-flat.pdf

Life sciences study design

All studies must disclose on these points even when the disclosure is negative.

Sample size	NA
Data exclusions	NA
Replication	NA
Randomization	NA
Blinding	NA

Reporting for specific materials, systems and methods

We require information from authors about some types of materials, experimental systems and methods used in many studies. Here, indicate whether each material, system or method listed is relevant to your study. If you are not sure if a list item applies to your research, read the appropriate section before selecting a response.

Materials & experimental systems

n/a	Involved in the study	
☒	☐	Antibodies
☒	☐	Eukaryotic cell lines
☒	☐	Palaeontology and archaeology
☐	☒	Animals and other organisms
☒	☐	Clinical data
☒	☐	Dual use research of concern
☒	☐	Plants

Methods

n/a	Involved in the study	
☒	☐	ChIP-seq
☒	☐	Flow cytometry
☒	☐	MRI-based neuroimaging

Animals and other research organisms

Policy information about studies involving animals; ARRIVE guidelines recommended for reporting animal research, and Sex and Gender in Research

Laboratory animals	NA
Wild animals	NA
Reporting on sex	NA
Field-collected samples	NA
Ethics oversight	NA

Note that full information on the approval of the study protocol must also be provided in the manuscript.

Plants

Seed stocks	NA
Novel plant genotypes	NA
Authentication	NA
